# Partner choice does not predict prosociality across countries

**DOI:** 10.1017/ehs.2022.51

**Published:** 2022-11-21

**Authors:** Scott Claessens, Thanos Kyritsis

**Affiliations:** School of Psychology, University of Auckland, Auckland, New Zealand

**Keywords:** partner choice, relational mobility, cooperation, prosociality, cross-cultural

## Abstract

Why does human prosociality vary around the world? Evolutionary models and laboratory experiments suggest that possibilities for partner choice (i.e. the ability to leave unprofitable relationships and strike up new ones) should promote cooperation across human societies. Leveraging the Global Preferences Survey (*n =* 27,125; 27 countries) and the World Values Survey (*n =* 54,728; 32 countries), we test this theory by estimating the associations between relational mobility, a socioecological measure of partner choice, and a wide variety of prosocial attitudes and behaviours, including impersonal altruism, reciprocity, trust, collective action and moral judgements of antisocial behaviour. Contrary to our pre-registered predictions, we found little evidence that partner choice is related to prosociality across countries. After controlling for shared causes of relational mobility and prosociality – environmental harshness, subsistence style and geographic and linguistic proximity – we found that only altruism and trust in people from another religion are positively related to relational mobility. We did not find positive relationships between relational mobility and reciprocity, generalised trust, collective action or moral judgements. These findings challenge evolutionary theories of human cooperation which emphasise partner choice as a key explanatory mechanism, and highlight the need to generalise models and experiments to global samples.

## Graphical abstract

**Figure:**
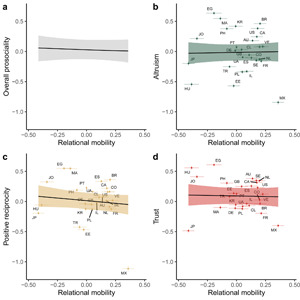

Humans are a uniquely prosocial species, and this prosociality is expressed in populations all around the world (Cronk et al., 2019). Yet, despite its ubiquity, there is also substantial global variation in prosociality, with some modern nation states expressing higher levels of cooperation than others (Dorrough & Glöckner, 2016; Romano et al., 2021; Van Doesum et al., 2021). What explains this variation in prosociality across countries?

One factor that could explain global variation in prosociality is differing possibilities for partner choice across countries. Here, ‘partners’ are defined as individuals that people socially interact with to provide mutual benefits (e.g. friends, neighbours, colleagues, mates). Theoretical models of partner choice show that when individuals can leave interactions with uncooperative partners and actively choose new interactions with cooperative partners, cooperation can evolve and be sustained (Aktipis, 2004, 2011; Enquist & Leimar, 1993; Roberts, 1998, 2020; Roberts et al., 2021). Partner choice allows for the assortative matching of cooperators, creating a market in which individuals use prosocial displays to compete for access to profitable social partnerships (Barclay, 2013, 2016). Thus, partner choice models predict that humans should be more prosocial and cooperative if they are able to leave unprofitable partnerships and freely choose new partnerships.

Laboratory and field evidence has begun to support theoretical models of partner choice. Experiments with economic games have shown that introducing partner choice causes people to cooperate more in social dilemmas (Barclay, 2004; Barclay & Raihani, 2016; Barclay & Willer, 2007; Sylwester & Roberts, 2010, 2013) and allowing for partner choice on dynamic social networks promotes assortative matching of cooperators (Jordan et al., 2013; Rand et al., 2011). Anthropological evidence also supports the role of partner choice in human cooperation, showing that people across a diverse range of societies selectively choose social partners with prosocial reputations, thereby encouraging prosociality (Bliege Bird & Power, 2015; Lyle & Smith, 2014; Smith & Apicella, 2020; Tognetti et al., 2014). For example, among the Aboriginal Australian Martu peoples, hunters with reputations as generous food sharers are more central in social networks and, as a result, receive more help from others (Bliege Bird & Power, 2015).

As well as predicting behaviour in the laboratory and in small-scale societies, partner choice models also predict that socioecological conditions favouring partner choice should promote prosociality in countries around the world. One recently developed socioecological variable that captures differing possibilities for partner choice is relational mobility (Yuki & Schug, 2012). Relational mobility captures ‘how much freedom and opportunity a society affords individuals to choose and dispose of interpersonal relationships based on personal preference’ (Thomson et al., 2018: 7521). In societies with low relational mobility, people do not actively choose their relationships and their social partners are relatively fixed. In contrast, in societies with high relational mobility, people actively choose who they interact with, creating dynamic social networks.

Societies with low levels of relational mobility are akin to classic partner control models in evolutionary game theory, where individuals are forced to interact for a fixed period (Axelrod & Hamilton, 1981). Partner control *can* promote prosocial behaviour, but only on the condition that individuals are able to reward their partners’ cooperative acts and effectively punish defection. In contrast, societies with high levels of relational mobility are akin to models of partner choice and biological markets (Barclay, 2013), which promote the evolution of cooperation under a potentially wider range of conditions than partner control models (Aktipis, 2004, 2011; Enquist & Leimar, 1993; Roberts, 1998, 2020; Roberts et al., 2021). Indeed, Barclay and Raihani (2016) found that people behave more prosocially when they can leave uncooperative partners compared with when they are forced to interact with them over fixed periods, even with the possibility of reciprocation and punishment.

We hypothesise, then, that people in higher relational mobility societies should express more prosocial behaviour and attitudes. Previous work has begun to test this hypothesis. For example, research has shown that people in higher relational mobility societies provide social support to others more frequently (Kito et al., 2017), have greater trust in strangers (Thomson et al., 2018) and are more likely to give gifts in romantic relationships (Komiya et al., 2019). Conversely, a recent meta-analysis found that people in higher relational mobility societies did not contribute more in incentivised social dilemma experiments (Spadaro et al., 2022). However, this previous work has focused on only a subset of possible measures of prosocial behaviours and attitudes: social support and cooperation in social dilemmas. Other kinds of prosociality predicted to increase under high levels of relational mobility include impersonal altruism, reciprocity, generalised trust, collective action and moral assessments of cheating behaviour. In addition, previous research has not studied the nature of the relationship between relational mobility and prosociality. While theoretical work has generally shown that partner choice promotes the evolution of cooperation, in some models too much partner choice is actually harmful for cooperation, because partner choice reduces interdependence with one's current partner (Barclay, 2020) and defectors can easily find new individuals to exploit (Aktipis, 2004). It is thus possible that the positive relationship between relational mobility and prosociality could have a ‘hump-backed’ shape, whereby relational mobility initially increases prosociality but too much relational mobility decreases it.

Here, we report the results of two pre-registered studies of the cross-national associations between relational mobility, our socioecological proxy for partner choice, and a range of prosocial behaviours and attitudes. In Study 1, we leveraged data from the Global Preferences Survey (Falk et al., 2018), a cross-national study of social preferences including impersonal altruism, positive reciprocity and generalised trust. We focused on these particular measures of prosociality because altruistic, reciprocal and trusting behaviours have been shown to reflect a single behavioural construct dubbed the ‘cooperative phenotype’ in previous work (Peysakhovich et al., 2014). All three of these behaviours are predicted to increase under higher levels of partner choice: altruistic and reciprocal prosocial behaviours become useful as signals of cooperative intent for potential partners, especially when broadcast publicly, and levels of trust thus increase along with levels of prosociality in the population. In Study 2, we used variables from the World Values Survey (Inglehart et al., 2014) measuring collective action, moral assessments of cheating behaviour and trust, which additionally capture people's prosocial contribution to social dilemmas and willingness to uphold prosocial moral norms.

Across both studies, we linked these prosociality data to relational mobility scores from a previous international survey (Thomson et al., 2018). Based on existing theory and literature, we pre-registered for both studies that we would find positive linear relationships between relational mobility and prosocial behaviours and attitudes: as relational mobility increases around the world, so should prosociality (https://osf.io/e528t/). In addition to our pre-registered analyses, we also explored potential non-linear relationships between relational mobility and prosocial behaviour and attitudes.

## Study 1

### Methods

#### Sample

In 2012, participants took part in the Global Preferences Survey (Falk et al., 2016, 2018), a large-scale study of economic decision-making across countries. This sample is unique in its measurement of social preferences with extensive global coverage. The full sample from the Global Preferences Survey contains 80,337 individuals from 76 countries. For the purposes of our study, we retained only participants from 27 countries that were also included in a 2018 multicountry study of relational mobility (Thomson et al., 2018). We also excluded participants who did not have data for any of the three main prosociality variables from the Global Preferences Survey: altruism, positive reciprocity and generalised trust. This resulted in a final sample of 27,125 individuals (15,107 female; mean age = 45.95 years, SD = 17.96 years). The countries retained in the final sample were Australia, Brazil, Canada, Chile, Colombia, Egypt, Estonia, France, Germany, Hungary, Israel, Japan, Jordan, Mexico, Morocco, the Netherlands, the Philippines, Poland, Portugal, South Korea, Spain, Sweden, Turkey, Ukraine, the UK, the USA and Venezuela (Supplementary Material, Figure S1).

The Global Preferences Survey was conducted as part of the 2012 Gallup World Poll (https://www.gallup.com/analytics/318875/global-research.aspx). The Gallup World Poll is conducted either via telephone or via face-to-face interview. For telephone interviews, nationally representative samples were achieved through the use of random-digit dialling or nationally representative lists of phone numbers. For face-to-face interviews, nationally representative samples were achieved through the use of a random route procedure within primary sampling units stratified by geography and/or population size.

#### Measures

*Prosociality.* Participants in the Global Preferences Survey were asked a series of self-report questions that measure the following social preferences: altruism, generalised trust, positive reciprocity, negative reciprocity, risk-taking and patience. For the purposes of our study, we focused on the altruism, trust and positive reciprocity items (for raw country-level data, see Supplementary Material, Table S1). Negative reciprocity was not studied, as previous factor analyses have shown that punitive behaviour forms a separate latent variable distinct from cooperation (Peysakhovich et al., 2014).

Altruism was measured by two items: a hypothetical charitable donation (‘Imagine the following situation: Today you unexpectedly received 1000 euros. How much of this amount would you donate to a good cause?’) and willingness to unconditionally donate to charity (‘How willing are you to give to good causes without expecting anything in return?’). Trust was measured by a single item: agreement with the statement ‘I assume that people have only the best intentions’. Positive reciprocity was measured by two items: stating the price of a hypothetical thank-you gift the participant would give to a stranger who helped them, and agreement with the statement ‘When someone does me a favour I am willing to return it’. These items have been shown to reliably predict altruistic, trusting, and reciprocal behaviour in incentivised economic decision-making experiments (Falk et al., 2016). These items also have metric invariance across countries (Supplementary Material, Table S2).

*Relational mobility.* We related measures of prosociality from the Global Preferences Survey to country-level relational mobility latent scores (Thomson et al., 2018). Country-level data on relational mobility were retrieved from a separate multicountry study (Thomson et al., 2018), in which 16,939 participants across 39 countries were contacted via an online survey between 2014 and 2016. We leveraged these data since they provide valid and reliable indicators of relational mobility across multiple countries. Country-level relational mobility latent scores were estimated from self-report ratings of the relational mobility of participants’ immediate societies, from a previously validated scale (Yuki et al., 2007). Measurement invariance analyses have shown that the scale has partial scalar invariance across countries. Positive correlations with related variables, like job mobility and number of new acquaintances, also indicate that the scale has high convergent validity (Thomson et al., 2018).

**Figure 1.**
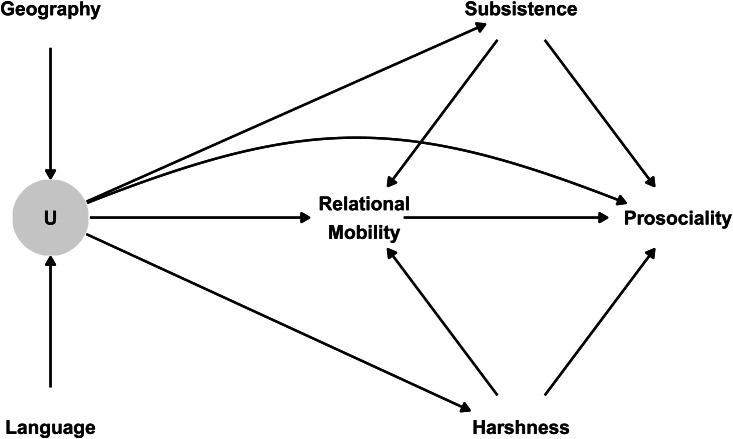
Directed acyclic graph of the causal model justifying the inclusion of covariates in our statistical models. Thomson et al. (2018) show that environmental harshness and subsistence style are antecedents of relational mobility, but other evidence also suggests that environmental harshness and subsistence style directly affect prosociality (Cronk et al., 2019; Talhelm et al., 2014). Environmental harshness and subsistence style are thus third variables that confound the direct path from relational mobility to prosociality. Moreover, all four of these variables are confounded by unmeasured factors (U), such as ecology, climate, institutions and norms. We cannot directly condition on unmeasured factors, but since these factors are themselves predicted by geographic and linguistic proximity between countries, we can account for them by allowing countries to covary according to geographic and linguistic proximity.

*Control variables.* In addition to our main variables, we also included several control variables in our regressions. These control variables are justified by a causal model in which both relational mobility and prosociality are jointly affected by various confounds (see Figure 1).

First, we controlled for environmental harshness and subsistence style. These two variables were retrieved from the same multicountry study of relational mobility (Thomson et al., 2018). Environmental harshness was a composite measure of seven indicators of historical and ecological threats: (1) history of territorial threats; (2) demanding geoclimate; (3) historical pathogen prevalence; (4) tuberculosis incidence; (5) disaster vulnerability; (6) population density in 1500; and (7) daily fat supply (reversed). Subsistence style was an index that represented the amount of area harvested with wheat, minus the percentage of pasture land for herding, plus the amount of harvested area devoted to rice farming, creating a continuum from relatively mobile and independent subsistence to more settled and interdependent subsistence. Thomson et al. (2018) argue that these country-level characteristics are key antecedents of relational mobility. Additional evidence suggests that these variables also affect prosociality (Cronk et al., 2019; Talhelm et al., 2014). These variables are thus shared causes that could confound the direct relationship between relational mobility and prosociality. We statistically conditioned on both environmental harshness and subsistence style to remove this confounding.

Second, we controlled for geographic and linguistic proximity between countries. Countries that are close to one another and share common cultural ancestors are likely to be more similar to one another, owing to similar ecologies, climates, institutions and norms (see Figure 1). To account for these unmeasured confounds, we allowed countries to covary according to geographic and linguistic proximity in our models. Geographic proximity was calculated as the inverse of the logged geodesic distance between country capital cities (data from the R package *maps*, Brownrigg, 2018) using the R package *geosphere* (Hijmans, 2019). Linguistic proximity between two countries was calculated as the cultural proximity between all languages spoken within those countries, weighted by speaker percentages (Eberhard et al., 2018; Hammarström et al., 2017); see the Supplementary Methods for more details.

#### Statistical analysis

To estimate the cross-national relationships between prosociality and relational mobility, we fitted pre-registered Bayesian multilevel regression models to the data (https://osf.io/e528t/). We analysed the data in long format, with multiple prosociality measures per participant (*n =* 80,885). The outcome variable was the score for the particular prosociality measure. The country-level predictor variable was the relational mobility latent score, with latent standard deviations included in the model to account for measurement error. We included random intercepts for participants and countries, and random intercepts and slopes for prosociality measures (altruism, trust and positive reciprocity; see Supplementary Methods). This multilevel structure deals with the fact that some countries have more observations than others, weighting the population-level estimates accordingly.

In order to systematically compare the various effects of our variables and controls, we fitted several models: (1) an intercept-only model; (2) a model including relational mobility as a predictor; (3) a model additionally controlling for environmental harshness and subsistence type; and (4) a model including controls and a quadratic effect of relational mobility. In all models, we allowed country random intercepts to covary according to geographic and linguistic proximity. Power analysis simulations revealed that the model with controls would be able to detect a medium effect of relational mobility (*β* = 0.28) with 83% power (Supplementary Material, Table S3). We used approximate leave-one-out cross-validation to compare models (Vehtari et al., 2017).

All analyses were conducted in R v4.0.2. (R Core Team, 2020). The *brms* package was used for Bayesian multilevel modelling (Bürkner, 2017). We used weakly informative priors and all models converged normally (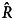 = 1). The *loo* package was used to compute approximate leave-one-out cross-validation scores (Vehtari et al., 2017). Visualisations were produced using the *ggplot2* (Wickham, 2016) and *cowplot* (Wilke, 2019) packages. The manuscript was reproducibly generated using the *targets* (Landau, 2021) and *papaja* (Aust & Barth, 2020) packages.

### Results and discussion

**Figure 2.**
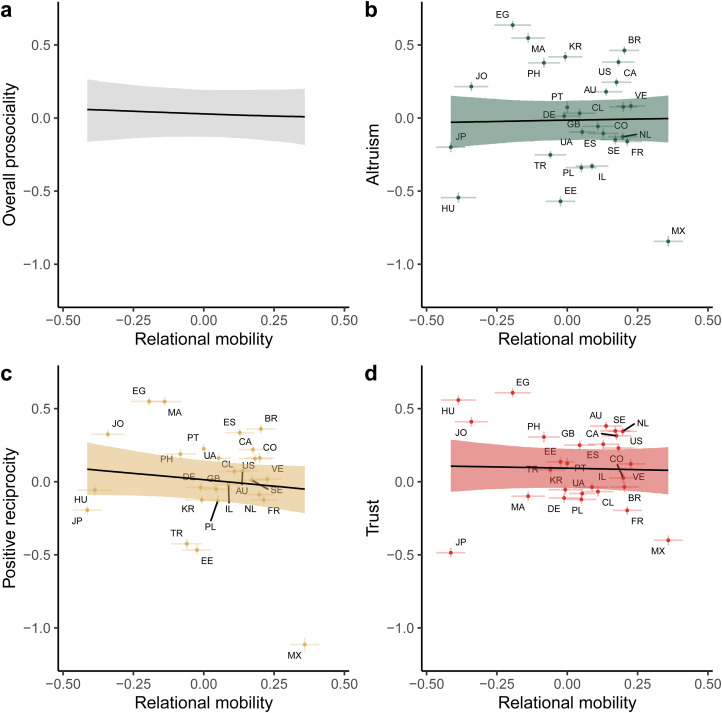
Posterior predictions from a Bayesian multilevel regression predicting prosocial preferences from country-level relational mobility, without control variables. (a) The overall effect of relational mobility on prosociality. (b–d) The individual effects of relational mobility on altruism, positive reciprocity and generalised trust. Lines and shaded areas indicate median posterior regression lines and 95% credible intervals. Points indicate average prosociality levels and relational mobility scores for each of the 27 countries, with error bars representing ±1 standard error. Letters represent country ISO codes.

Model comparison revealed that adding relational mobility as a predictor of prosocial preferences did not improve model fit over a null intercept-only model (difference in expected log predictive density = 7.74, standard error = 6.66). The median posterior slope for relational mobility predicting overall prosocial preferences was −0.03, 95% credible interval [−0.22 0.16] (Figure 2). Incorporating item random effects further revealed that relational mobility did not predict altruism (median posterior slope = 0.04, 95% CI [−0.26 0.30]), positive reciprocity (median posterior slope = −0.17, 95% CI [−0.48 0.09]) or generalised trust (median posterior slope = −0.03, 95% CI [−0.33 0.23]).

**Figure 3.**
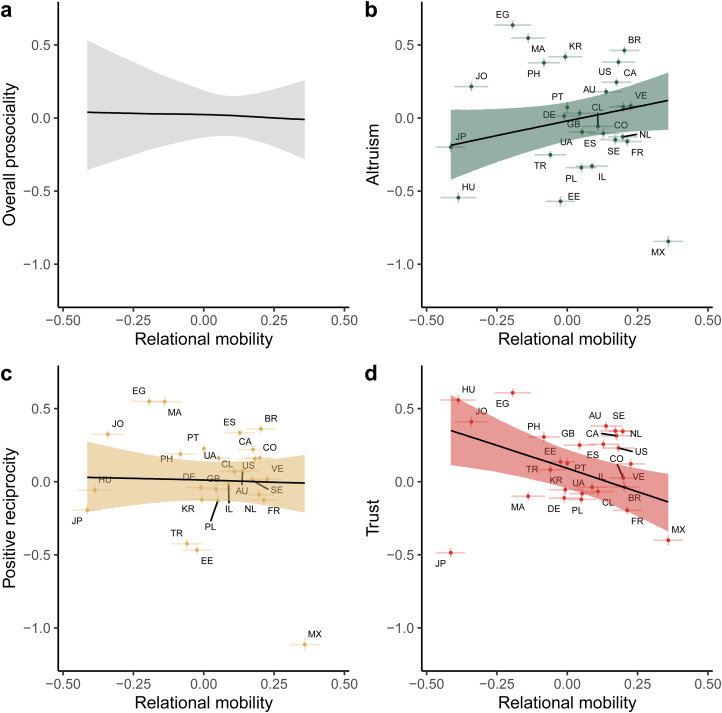
Posterior predictions from a Bayesian multilevel regression predicting prosocial preferences from country-level relational mobility, controlling for environmental harshness and subsistence style. (a) The overall effect of relational mobility on prosociality. (b–d) The individual effects of relational mobility on altruism, positive reciprocity, and generalised trust. Lines and shaded areas indicate median posterior regression lines and 95% credible intervals. Points indicate average prosociality levels and relational mobility scores for each of the 27 countries, with error bars representing ±1 standard error. Letters represent country ISO codes.

We also included two additional predictors as control variables: environmental harshness and subsistence style. Model comparison revealed that additionally conditioning on both environmental harshness and subsistence style improved model fit over a model containing only relational mobility (difference in expected log predictive density = 527.58, standard error = 32.75). The median posterior slope for relational mobility predicting overall prosocial preferences was −0.02, 95% credible interval [−0.20 0.17] (Figure 3). Incorporating random effects further revealed that relational mobility now slightly positively predicted altruism (median posterior slope = 0.40, 95% CI [−0.07 0.83]), did not predict positive reciprocity (median posterior slope = −0.05, 95% CI [−0.52 0.38]) and *negatively* predicted generalised trust (median posterior slope = −0.63, 95% CI [−1.11 −0.20]). The slight relationship between relational mobility and impersonal altruism is in line with our pre-registered hypothesis, but the negative relationship between relational mobility and generalised trust contradicts previous research suggesting that relational mobility is positively related to trust in others (Thomson et al., 2018; Yuki et al., 2007). There was no quadratic effect of relational mobility in the model including controls (Supplementary Material, Table S4).

There are several possible explanations for these mixed results. First, over half of our sample of countries were from Western Europe and North America, where relational mobility is higher than average. This does not leave much variation to detect associations, especially with a small sample size of 27 countries. Second, only a small set of prosociality measures were available in the Global Preferences Survey, limited to charitable donations, exchanges of gifts and favours, and generalised trust. As such, this dataset did not cover other important aspects of prosociality, such as prosocial contributions to social dilemmas and willingness to uphold prosocial norms.

In order to investigate whether these factors could explain our results, we conducted a second study with a different dataset. In Study 2, we leveraged data from the World Values Survey (Inglehart et al., 2014), a multicountry self-report study of values and attitudes. This study has global coverage and includes items measuring a wide variety of prosocial behaviours and attitudes. We were able to link data from 32 countries to country-level data on relational mobility, expanding our sample size and including additional Asian countries. We hypothesised that individuals from countries with higher relational mobility would be more likely to belong to humanitarian and charitable organisations, our measure of collective action and prosocial contribution to social dilemmas, and more likely to report that violations of prosocial norms are morally unjustifiable. Both of these are indirect measures of cooperative and prosocial behaviours that could feasibly provide signals of cooperative intent in biological markets. Repeating the prediction from our first study, we also hypothesise that individuals from countries with higher relational mobility will show higher levels of trust in others.

## Study 2

### Methods

#### Sample

Between 2017 and 2020, participants completed either the seventh wave of the World Values Survey or the fifth wave of the European Values Survey. The full sample size from these combined waves was 135,000 participants from 81 countries. For the purposes of our study, we retained only participants from 32 countries that were also included in Thomson et al. (2018). This resulted in a final sample of 54,728 individuals (29,141 female; mean age = 47.49 years, SD = 17.33 years). The countries retained in the final sample were Australia, Brazil, Canada, Chile, Colombia, Egypt, Estonia, France, Germany, Hong Kong, Hungary, Japan, Jordan, Lebanon, Malaysia, Mexico, the Netherlands, New Zealand, the Philippines, Poland, Portugal, Puerto Rico, Singapore, South Korea, Spain, Sweden, Taiwan, Tunisia, Turkey, Ukraine, the UK, and the USA (Supplementary Material, Figure S2).

The World Values Survey and the European Values Survey are conducted mainly via face-to-face interviews. The surveys contact a minimum sample of 1200 participants per country. All samples are representative of the population aged 18 and over, via full probability or a combination of probability and stratified sampling methods.

#### Measures

*Prosociality.* Participants in both the World Values Survey and the European Values Survey answer a range of self-report questions on social values, societal wellbeing, trust, economic values, religion, politics and ethics. For the purposes of our study, we highlighted several variables as measures of cooperation, trust and prosociality (for raw country-level data, see Supplementary Material, Table S5). The first variable captures cooperation via collective action: ‘Are you a member of a charitable or humanitarian organisation?’ For a similar interpretation of this variable, see Jacquet et al. (2021). The second variable captures generalised trust: ‘Generally speaking, would you say that most people can be trusted or that you need to be very careful in dealing with people?’ The third set of variables captures levels of trust in specific groups of people, namely family, neighbourhood, personal acquaintances, people the respondent has met for the first time, people of another religion and people of another nationality. The fourth set of variables captures the justifiability of different self-interested moral trangressions, including claiming unentitled government benefits, avoiding a fare on public transport, cheating on taxes and someone accepting a bribe. Both the set of items measuring trust in different groups and the set of items measuring moral justifiability for different moral transgressions have metric invariance across countries (Supplementary Material, Tables S6 and S7).

*Relational mobility and control variables.* As in Study 1, we related prosociality measures to country-level relational mobility latent scores (Thomson et al., 2018). We also controlled for the same measures of environmental harshness and subsistence style, and allowed countries to covary according to the same measures of geographic and linguistic proximity.

#### Statistical analysis

To estimate cross-national relationships, we fitted pre-registered Bayesian multilevel models to the data (https://osf.io/e528t/). For the charitable organisation and generalised trust variables, we fitted logistic regression models for binary data with random intercepts for countries. For trust in specific groups and justifiability of moral transgressions, we converted the data to long format, reversed the outcome variable such that higher values reflect higher levels of prosociality, and fitted cumulative link regression models for ordinal data. In these models, we included random intercepts for individuals and countries, and random intercepts and slopes for groups/moral transgressions (see Supplementary Methods).

As described in Study 1, we included measurement error on the relational mobility latent scores and accounted for spatial and cultural non-independence between countries with correlated random intercepts. We additionally fitted models that controlled for environmental harshness and subsistence style and included a quadratic effect of relational mobility. Power analysis simulations revealed that the models with controls would be able to detect small-to-medium effects of relational mobility with roughly 80% power (Supplementary Material, Table S3). All analyses were conducted in R v4.0.2. (R Core Team, 2020).

### Results and discussion

**Figure 4.**
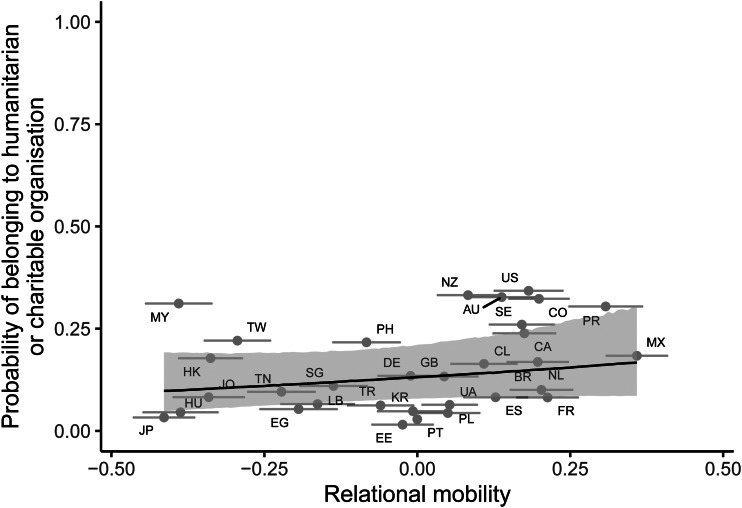
Posterior predictions from a Bayesian multilevel logistic regression predicting charitable organisation membership from country-level relational mobility, without controls. The line and shaded area indicate the median posterior regression line and 95% credible intervals. Points indicate the proportion of individuals belonging to charitable organisations on the *y*-axis and relational mobility scores on the *x*-axis, for each of the 32 countries, with error bars representing ±1 standard error. Letters represent country ISO codes.

For our measure of cooperation and collective action – charitable organisation membership – model comparison revealed that adding relational mobility as a predictor improved model fit over a null intercept-only model (difference in expected log predictive density = 43.06, standard error = 0.99). The posterior log odds slope for relational mobility predicting charitable organisation membership was in the expected direction, but the 95% credible interval included zero (median posterior slope = 0.80, 95% CI [−0.58 2.10]; Figure 4). The 95% credible interval continued to include zero after controlling for environmental harshness and subsistence type (median posterior slope = 0.20, 95% CI [−1.30 1.73]; Supplementary Material, Figure S3). There was no quadratic effect of relational mobility on charitable organisation membership (Supplementary Material, Table S4).

**Figure 5.**
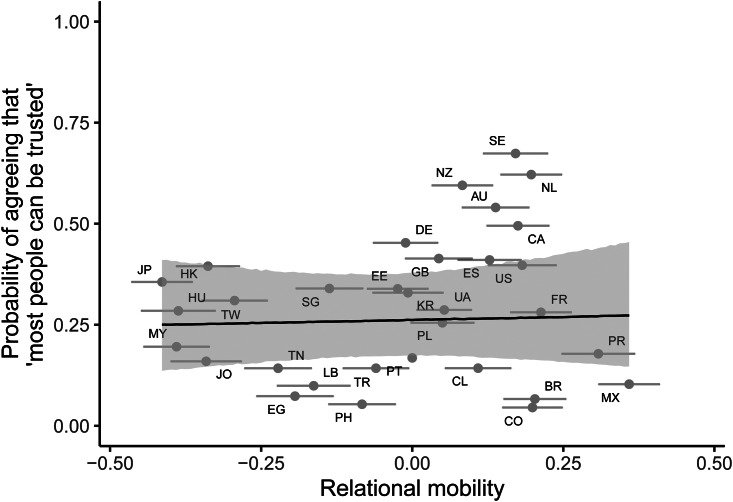
Posterior predictions from a Bayesian multilevel logistic regression predicting generalised trust from country-level relational mobility, without controls. The line and shaded area indicate the median posterior regression line and 95% credible intervals. Points indicate the proportion of individuals stating that ‘most people can be trusted’ on the *y*-axis and relational mobility scores on the *x*-axis, for each of the 32 countries, with error bars representing ±1 standard error. Letters represent country ISO codes.

For generalised trust, model comparison revealed that adding relational mobility as a predictor improved model fit over a null intercept-only model (difference in expected log predictive density = 32.21, standard error = 0.99). The 95% credible interval for the posterior log odds slope for relational mobility predicting generalised trust included zero (median posterior slope = 0.16, 95% CI [−1.29 1.57]; Figure 5). The 95% credible interval continued to include zero after controlling for environmental harshness and subsistence type (median posterior slope = 0.11, 95% CI [−1.32 1.62]; Supplementary Material, Figure S4). There was no quadratic effect of relational mobility on generalised trust (Supplementary Material, Table S4).

**Figure 6.**
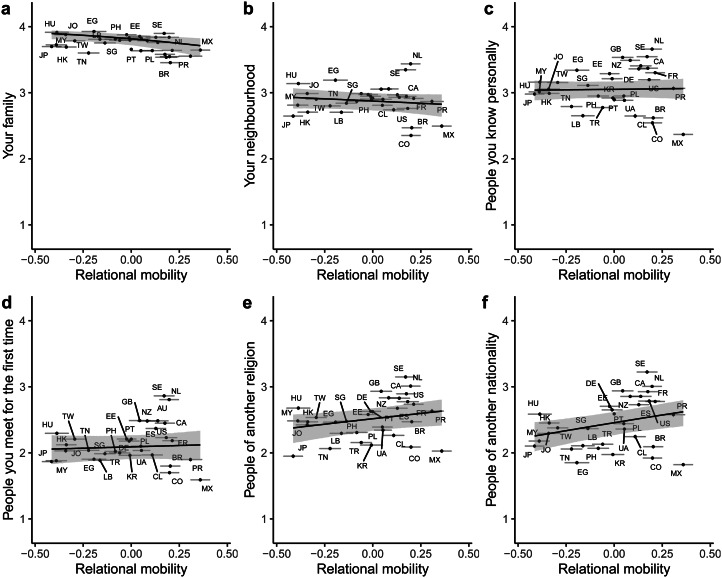
Posterior predictions from a Bayesian multilevel ordinal regression predicting trust in specific groups from country-level relational mobility, without controls. Higher numbers on the *y*-axis indicate higher levels of trust. Lines and shaded areas indicate median posterior regression lines and 95% credible intervals. Points indicate average trust and relational mobility scores for each of the 32 countries, with error bars representing ±1 standard error. Letters represent country ISO codes.

For trust in specific groups (Figure 6), random slopes revealed that relational mobility was *negatively* related to trust in family (median posterior slope = −1.59, 95% CI [−2.55 −0.63]). Relational mobility was unrelated to trust in one's neighbourhood (median posterior slope = −0.56, 95% CI [−1.52 0.41]), trust in people one knows personally (median posterior slope = 0.15, 95% CI [−0.81 1.09]) and trust in people one meets for the first time (median posterior slope = 0.25, 95% CI [−0.71 1.20]). Relational mobility was positively related to trust in people of another religion (median posterior slope = 1.02, 95% CI [0.06 1.98]) and trust in people of another nationality (median posterior slope = 1.45, 95% CI [0.49 2.39]). Only the relationship between relational mobility and trust in people of another religion was attenuated after controlling for environmental harshness and subsistence style (median posterior slope = 0.51, 95% CI [−0.48 1.48]; Supplementary Material, Figure S5). Quadratic effects revealed non-linear relationships between relational mobility and trust in family, people one knows personally and people of another nationality, but the effects were small (Supplementary Material, Table S4; Supplementary Material, Figure S6).

**Figure 7.**
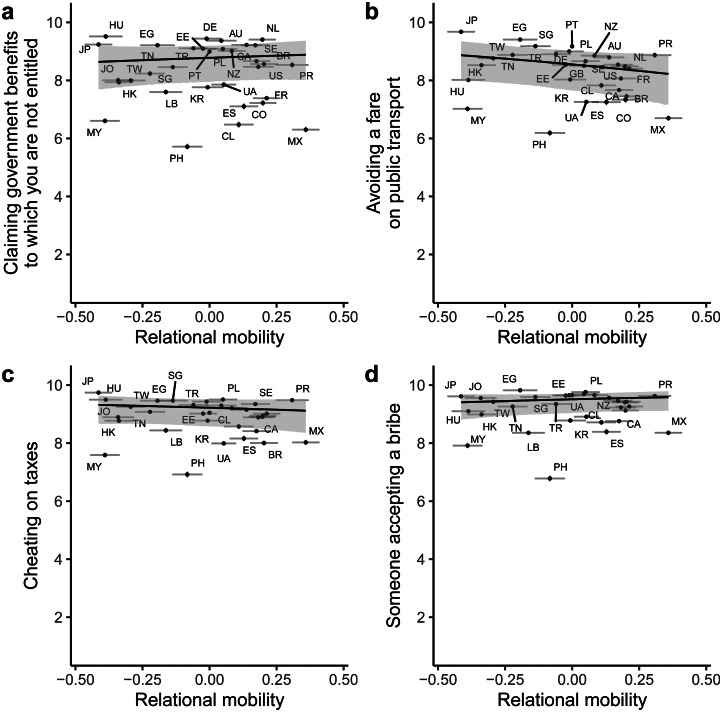
Posterior predictions from a Bayesian multilevel ordinal regression predicting moral justifiability of different scenarios from country-level relational mobility, without controls. Higher numbers on the *y*-axis indicate *lower* justifiability ratings for each scenario, such that higher values reflect higher levels of prosociality. Lines and shaded areas indicate median posterior regression lines and 95% credible intervals. Points indicate average justifiability (reversed) and relational mobility scores for each of the 32 countries, with error bars representing ±1 standard error. Letters represent country ISO codes.

For moral justifiability of self-interested moral transgressions, model comparison revealed that adding relational mobility as a predictor improved model fit over a null intercept-only model (difference in expected log predictive density = 324.53, standard error = 28.62; Figure 7). In this model, random slopes revealed that relational mobility was unrelated to self-reported justifiability for all four scenarios: claiming government benefits to which one is not entitled (median posterior slope = 0.39, 95% CI [−0.75 1.53]), avoiding a fare on public transport (median posterior slope = −0.91, 95% CI [−2.06 0.24]), cheating on taxes (median posterior slope = −0.42, 95% CI [−1.57 0.70]) and someone accepting a bribe (median posterior slope = 0.56, 95% CI [−0.61 1.70]). These results remained unchanged after controlling for environmental harshness and subsistence style (Supplementary Material, Figure S7). Quadratic effects revealed non-linear relationships between relational mobility and two moral transgressions, claiming government benefits and cheating on taxes, but the effects were small (Supplementary Material, Table S4; Supplementary Material, Figure S8).

Overall, contrary to our pre-registered hypotheses, we found that relational mobility was unrelated to collective action (operationalised as charitable organisation membership), generalised trust and moral justifiability ratings for self-interested behaviours. Relational mobility was also unrelated to trust in most specific groups, although we did find that relational mobility negatively predicted trust in family and positively predicted trust in people of another religion and nationality. This ‘scope of trust’ effect, whereby relational mobility is associated with lower trust in closer contacts but greater trust in more distant contacts, is an interesting feature of the construct that aligns with previous work (Thomson et al., 2018).

## General discussion

Across two pre-registered cross-national studies, we found little evidence to suggest that partner choice via relational mobility is positively associated with prosociality around the world. In our first study, we initially found no relationships between relational mobility and altruism, positive reciprocity or trust. Only when we controlled for environmental harshness and subsistence style did we find that relational mobility negatively predicted trust and slightly positively predicted altruism. In our second study, we found no relationships between relational mobility and collective action, generalised trust or moral judgements of antisocial behaviour. Relational mobility was also unrelated to trust in most specific groups, although we found that relational mobility did negatively predict trust in family and positively predict trust in people of another religion and nationality.

Why did we not find the expected relationships between relational mobility and prosociality for most measures? One might argue that relational mobility is not an adequate measure of the kinds of partner choice implemented in theoretical models of cooperation or laboratory experiments. We would contest this view. Relational mobility is explicitly defined as a construct that quantifies ‘variance in partner choice in human societies’ akin to biological markets (Thomson et al., 2018: 7521). In the relational mobility scale, people are asked about their immediate society, including friends, acquaintances, colleagues, and neighbours, and whether these people can ‘leave [current relationships] for better ones’ and ‘choose … the people they interact with’. These are the exact same opportunities afforded to agents in partner choice models and participants in partner choice experiments. For example, the Walk Away strategy has the ability to choose new interaction partners and leave those interaction partners if they defect (Aktipis, 2004).

Others might argue that our measures of prosociality lacked construct validity or were not suitable for cross-country comparisons. We acknowledge that our outcome variables were self-reported rather than behavioural measures that in some cases (e.g. charitable membership organisation) mapped only loosely onto the construct of interest. This was largely unavoidable using secondary data. However, the self-report measures of prosociality from the Global Preferences Survey were generated based on their strong positive relationships with prosocial behaviour in incentivised economic games, and yet the evidence with these measures remained mixed. Moreover, we found that all of our outcome variables exhibited metric invariance (i.e. invariant factor loadings) across countries, suggesting that participants attributed the same meanings to the constructs around the world. Although we did not find scalar invariance (i.e. invariant item intercepts) for these measures, researchers have suggested that this level of invariance is an overly strict threshold for cross-cultural comparisons of many groups (Selig et al., 2008) and does not necessarily imply incomparability of measures across groups (Welzel et al., 2021). Future work should assess the comparability and comprehension of survey measures of prosociality across countries.

It is also unlikely that our null results arose from a non-linear relationship between relational mobility and prosociality. Some theoretical models find that extreme levels of partner choice actually become harmful for the evolution of cooperation (Aktipis, 2004). Under this view, relational mobility might initially promote prosocial behaviour but reduce it again at high levels, masking any simple linear relationship between relational mobility and prosociality. However, our statistical models with quadratic terms revealed no pronounced ‘hump-shaped’ relationships between relational mobility and prosociality. Instead, the 95% credible intervals for most quadratic effects included zero.

Instead of arising as artefacts of operationalisations, self-report measures or potential non-linear effects, we are confident that our findings reflect a true null relationship between relational mobility and prosociality. Across two studies, we leveraged large samples in a multilevel design, allowing us to make claims about individual-level psychology in socioecological context. We used a wide variety of prosociality measures. We explicitly mapped out a causal diagram and controlled for various sources of confounding in our statistical models, including geographic and cultural non-independence, an issue that is largely ignored in cross-national studies and can create spurious inferences (Bromham et al., 2018; Claessens & Atkinson, 2022). We also directly modelled measurement error on the relational mobility variable, since this country-level variable was a factor score that was itself measured imperfectly (Thomson et al., 2018). With these methodological strengths, we found that relational mobility was not reliably related to prosociality, a null result that is line with a previous meta-analytic study (Spadaro et al., 2022).

Our findings build on and contrast with previous work. Thomson et al. (2018) found that relational mobility was positively related to trust in strangers. Supporting this link, we found a ‘scope of trust’ effect, whereby relational mobility negatively predicted trust in close contacts (family members) and positively predicted trust in distant contacts (people of other religions and nationalities). This pattern of associations reveals that, with multiple groups of increasing social distance, relational mobility scales up people's circles of trust beyond close kin. This finding is in line with recent models of partner choice, fitness interdependence, and anonymous helping (Barclay, 2020). These models show that in environments where partners are more easily replaced, individuals become less interdependent with their existing partners, thus reducing the amount of prosociality towards close contacts and increasing the amount of prosociality towards distant contacts.

However, previous research has also shown that relational mobility is positively related to generalised trust, willingness to help close friends, social support towards close friends, and gift-giving in romantic relationships (Kito et al., 2017; Thomson et al., 2018; Yuki et al., 2007; Yuki & Schug, 2012). In contrast to this previous research, we found that relational mobility is either unrelated or negatively related to generalised trust, and is also unrelated to willingness to return a favour and gift-giving, as well as a host of other prosocial behaviours and attitudes. These differences in results may have arisen from differences in analytic strategies. For example, Thomson et al. (2018) conducted country-level correlations, and only found a relationship between relational mobility and generalised trust when excluding Hungary and Latin American countries (*N* = 27). In contrast, we conducted individual-level multilevel models with measurement error and controls for statistical non-independence between countries.

Taken together, these null findings challenge previous theoretical and empirical studies suggesting that partner choice promotes prosociality and cooperation in humans. Theoretical models show that introducing the possibility of partner choice creates conditions that favour the evolution of cooperation (Aktipis, 2004, 2011; Enquist & Leimar, 1993; Roberts, 1998, 2020; Roberts et al., 2021). Laboratory and field work also suggest that partner choice, over and above simple reputational effects, encourages forms of competitive prosociality as people endeavour to be chosen for profitable partnerships (Barclay, 2004; Barclay & Raihani, 2016; Barclay & Willer, 2007; Bliege Bird & Power, 2015; Sylwester & Roberts, 2010, 2013). Yet our findings suggest that cross-national variation in prosociality is not well explained by differences in possibilities for partner choice.

It is possible that relational mobility does affect prosocial behaviour and attitudes, but at a more local scale. Our biased sample of countries reflects a set of large-scale modern industrialised societies which are uncharacteristic of most of human history. Large-scale societies mostly promote and enforce prosociality through formal centralised institutions (e.g. courts, laws). In small-scale societies, in contrast, prosociality is more often promoted through local social norms that guide partner choice, reputation and reciprocity (Glowacki & Lew-Levy, 2022). This could explain why our cross-national results differ from those from previous field studies which measure partner choice in small-scale societies. To test this possibility, future research should employ the relational mobility self-report measures in a wider variety of societies with different social scales and cultural backgrounds, ideally including non-Western and small-scale societies.

It is also possible that people in low relational mobility nations are just as prosocial as people in high relational mobility nations, but this prosociality is achieved in different ways. Partner control models, such as the iterated Prisoner's Dilemma (Axelrod & Hamilton, 1981), show that strategies can successfully promote cooperation in fixed interactions if they cooperate conditionally and punish non-cooperation (e.g. tit-for-tat strategies). Likewise, repeatedly interacting individuals in low relational mobility nations might use these same mechanisms to encourage prosociality in their own ways. As a result, it may be that countries around the world have all reached some equilibrium level of prosociality, either through partner control or partner choice mechanisms. To test this idea, future research should measure not levels of prosociality *per se*, but rather the mechanisms by which they achieve that level of prosociality. For example, we might predict that social interactions in low relational mobility nations should be characterised by conditional cooperation, quick rescindments of cooperation from defectors, and high levels of peer-to-peer punishment, rather than leaving to search for alternative partners.

In sum, we found little evidence that partner choice, proxied as relational mobility, is related to cross-national variation in prosociality around the world. These findings challenge evolutionary theories that seek to explain why human cooperation has flourished and been maintained around the world. They also highlight the need to connect theoretical models and tightly controlled experiments with global samples to make generalisable claims about human behaviour.

## Data Availability

All data are available on the Open Science Framework: https://osf.io/e528t/

## Research transparency and reproducibility

All data and code to reproduce the statistical analyses in this manuscript can be found on the Open Science Framework: https://osf.io/e528t/

## Supplementary material

For supplementary material accompanying this paper visit https://doi.org/10.1017/ehs.2022.51. click here to view supplementary material
