# Supplementary material for: Partner choice does not predict prosociality across countries
Source: Evol Hum Sci. 2022 Nov 21;4:e54. doi: 10.1017/ehs.2022.51 (PMC10426035; doi:10.1017/ehs.2022.51)
Supplement: Supplementary file 1 [file S2513843X22000512sup001.pdf]

Supplementary Material

Supplementary Methods

Calculating linguistic distances between nations. Linguistic distance between two countries was calculated as the cultural proximity between all languages spoken within those countries, weighted by speaker percentages. We acquired cultural proximity data by combining the language family trees provided by Glottolog v3.0 (Hammarström et al., 2017) into one global language tree (undated and unresolved). We calculated cultural proximity s between two languages j and k as the distance (in number of nodes traversed) of their most recent common ancestor i to the root of the tree, through the formula:

$$s_{jk} = \frac{n_r - n_i}{n_r}$$

where n_r is the maximum path length (in number of nodes traversed) leading to the pan-human root r, and n_i is the maximum path length leading to node i. We then combined these proximities with speaker data from Ethnologue 21 (Eberhard et al., 2018) and compared every language spoken within those countries by at least 1 permille of the population, weighted by speaker percentages, through the formula:

$$w_{lm} = \Sigma\Sigma p_{lj} p_{mk} s_{jk}$$

where p_{lj} is the percentage of the population in nation l speaking language j, p_{mk} is the percentage of the population in nation m speaking language k, and s_{jk} is the proximity measure between languages j and k (Eff, 2008).

Bayesian multilevel models. In both Studies 1 and 2, we use Bayesian multilevel models to test our hypotheses. Below, we write out the formulae for the different models.

We focus on models that include relational mobility as the only predictor, but these can be generalised to include additional predictors as fixed effects.

In Study 1, we model prosociality as the outcome variable (Pro), relational mobility as the country-level predictor variable (Rel), random intercepts and slopes for different prosociality items in the Global Preferences Survey (item; altruism, positive reciprocity, and trust), and random intercepts for participants (part) and countries (country).

To deal with spatial and cultural non-independence between countries, we allow separate random intercepts for countries to covary according to geographic ($\mathbf{G}$) and linguistic ($\mathbf{L}$) proximity matrices. This is similar to the approach employed in phylogenetic general linear mixed models, which deal with the non-independent structure in model 'residuals' by including a pre-computed covariance matrix specifying the relationships between species (Villemereuil & Nakagawa, 2014; see also here). In addition to these random effects, we include a residual random intercept over countries to capture country-specific effects that are independent of geographic and linguistic relationships with other countries.

We also model relational mobility with measurement error by including standard deviations (Rel_{SD}) from observed latent variable means (Rel_{OBS}). This ensures that the uncertainty in the measurement of relational mobility from previous research is propagated into this model.

The model formula is as follows:

$$
\begin{aligned}
\text{Pro}_i &\sim \text{Normal}(\mu_i, \sigma) \\
\mu_i &= \alpha_i + \beta_i \text{Rel}_{\text{TRUE},i} \\
\text{Rel}_{\text{TRUE},i} &= \lambda + \kappa z_{\text{country}[i]} \\
\text{Rel}_{\text{OBS},i} &\sim \text{Normal}(\text{Rel}_{\text{TRUE},i}, \text{Rel}_{\text{SD},i}) \\
\alpha_i &= \bar{\alpha} + \alpha_{\text{item}[i]} + \alpha_{\text{part}[i]} + \alpha_{\text{G,country}[i]} + \alpha_{\text{L,country}[i]} + \alpha_{\text{R,country}[i]} \\
\beta_i &= \bar{\beta} + \beta_{\text{item}[i]} \\
\begin{bmatrix} \alpha_{\text{item}} \\ \beta_{\text{item}} \end{bmatrix} &\sim \text{MVNormal}\left(\begin{bmatrix} 0 \\ 0 \end{bmatrix}, \mathbf{S} \right) \\
\mathbf{S} &= \begin{pmatrix} \tau_\alpha & 0 \\ 0 & \tau_\beta \end{pmatrix} \mathbf{R} \begin{pmatrix} \tau_\alpha & 0 \\ 0 & \tau_\beta \end{pmatrix} \\
\alpha_{\text{part}} &\sim \text{Normal}(0, \tau_P) \\
\alpha_{\text{G,country}} &\sim \text{Normal}(0, \tau_G \mathbf{G}) \\
\alpha_{\text{L,country}} &\sim \text{Normal}(0, \tau_L \mathbf{L}) \\
\alpha_{\text{R,country}} &\sim \text{Normal}(0, \tau_R) \\
z_{\text{country}} &\sim \text{Normal}(0, 1) \\
\bar{\alpha}, \bar{\beta}, \lambda &\sim \text{Normal}(0, 0.1) \\
\mathbf{R} &\sim \text{LKJCorr}(1) \\
\kappa, \tau_\alpha, \tau_\beta, \tau_P, \tau_G, \tau_L, \tau_R, \sigma &\sim \text{Exponential}(5)
\end{aligned}
$$

where $\bar{\alpha}$ and $\bar{\beta}$ represent intercept and slope fixed effects, other α and β parameters represent random intercepts and slopes, τ parameters represent standard deviations for random effects, $\mathbf{R}$ represents the correlation matrix for the item random effects, σ represents the residual variance, and λ, κ, and z represent the mean, standard deviation, and standardised latent values for the relational mobility measurement error model.

As a formula suitable for the R package *brms*, the model is written as follows:

```
brm(
  formula = Pro ~ 0 + Intercept + me(RML, RML_SE, gr = iso) +
    (1 + RML | item) + (1 | id) + (1 | gr(iso1, cov = lingCov)) +
    (1 | gr(iso2, cov = geoCov)) + (1 | iso3),
  data = d,
  family = gaussian,
  prior = c(prior(normal(0, 0.1), class = b),
            prior(exponential(5), class = sd),
            prior(exponential(5), class = sigma),
            prior(normal(0, 0.1), class = meanme),
            prior(exponential(5), class = sdme)),
  data2 = list(lingCov = lingCov, geoCov = geoCov)
  )
```

In Study 2, we use two types of Bayesian multilevel model. To analyse binary data on charitable organisation membership (Org) and generalised trust (GenTru), we use multilevel logistic regression models with random intercepts for countries. As in Study 1, we allow country random intercepts to vary according to geographic and linguistic proximity, and we model measurement error on the relational mobility predictor.

$$
\begin{aligned}
\text{Org}_i/\text{GenTru}_i &\sim \text{Bernoulli}(p_i) \\
\text{logit}(p_i) &= \alpha_i + \beta\text{Rel}_{\text{TRUE},i} \\
\text{Rel}_{\text{TRUE},i} &= \lambda + \kappa z_{\text{country}[i]} \\
\text{Rel}_{\text{OBS},i} &\sim \text{Normal}(\text{Rel}_{\text{TRUE},i}, \text{Rel}_{\text{SD},i}) \\
\alpha_i &= \bar{\alpha} + \alpha_{\text{G,country}[i]} + \alpha_{\text{L,country}[i]} + \alpha_{\text{R,country}[i]} \\
\alpha_{\text{G,country}} &\sim \text{Normal}(0, \tau_G \mathbf{G}) \\
\alpha_{\text{L,country}} &\sim \text{Normal}(0, \tau_L \mathbf{L}) \\
\alpha_{\text{R,country}} &\sim \text{Normal}(0, \tau_R) \\
\lambda &\sim \text{Normal}(0, 0.1) \\
\kappa &\sim \text{Exponential}(5) \\
\bar{\alpha}, \beta, z_{\text{country}} &\sim \text{Normal}(0, 1) \\
\tau_G, \tau_L, \tau_R &\sim \text{Exponential}(2)
\end{aligned}
$$

where $\bar{\alpha}$ and β represent the intercept and slope fixed effects, other α parameters represent random intercepts, τ parameters represent standard deviations for random effects, and λ, κ, and z represent the mean, standard deviation, and standardised latent values for the relational mobility measurement error model.

In *brms*, this model is written as follows:

```
brm(
  formula = Org ~ 0 + Intercept + me(RML, RML_SE, gr = iso) +
    (1 | gr(iso1, cov = lingCov)) + (1 | gr(iso2, cov = geoCov)) +
    (1 | iso3),
  data = d, family = bernoulli,
  prior = c(prior(normal(0, 1), class = b),
            prior(exponential(2), class = sd),
            prior(normal(0, 0.1), class = meanme),
            prior(exponential(5), class = sdme)),
  data2 = list(lingCov = lingCov, geoCov = geoCov)
  )
```

To analyse ordinal data on trust in different groups (Trust) and moral justifiability of different antisocial behaviours (Just), we use multilevel cumulative link regression models with random intercepts and slopes for groups / behaviours (item), as well as random intercepts for participants and countries. Again, as in Study 1, we allow country random intercepts to vary according to geographic and linguistic proximity, and we model measurement error on the relational mobility predictor.

$$
\begin{aligned}
\text{Trust}_i/\text{Just}_i &\sim \text{Ordered-logit}(\phi_i, \zeta) \\
\phi_i &= \alpha_i + \beta_i \text{Rel}_{\text{TRUE},i} \\
\text{Rel}_{\text{TRUE},i} &= \lambda + \kappa z_{\text{country}[i]} \\
\text{Rel}_{\text{OBS},i} &\sim \text{Normal}(\text{Rel}_{\text{TRUE},i}, \text{Rel}_{\text{SD},i}) \\
\alpha_i &= \alpha_{\text{item}[i]} + \alpha_{\text{part}[i]} + \alpha_{\text{G,country}[i]} + \alpha_{\text{L,country}[i]} + \alpha_{\text{R,country}[i]} \\
\beta_i &= \bar{\beta} + \beta_{\text{item}[i]} \\
\begin{bmatrix} \alpha_{\text{item}} \\ \beta_{\text{item}} \end{bmatrix} &\sim \text{MVNormal}\left(\begin{bmatrix} 0 \\ 0 \end{bmatrix}, \mathbf{S} \right) \\
\mathbf{S} &= \begin{pmatrix} \tau_\alpha & 0 \\ 0 & \tau_\beta \end{pmatrix} \mathbf{R} \begin{pmatrix} \tau_\alpha & 0 \\ 0 & \tau_\beta \end{pmatrix} \\
\alpha_{\text{part}} &\sim \text{Normal}(0, \tau_P) \\
\alpha_{\text{G,country}} &\sim \text{Normal}(0, \tau_G \mathbf{G}) \\
\alpha_{\text{L,country}} &\sim \text{Normal}(0, \tau_L \mathbf{L}) \\
\alpha_{\text{R,country}} &\sim \text{Normal}(0, \tau_R) \\
z_{\text{country}} &\sim \text{Normal}(0, 1) \\
\zeta_j &\sim \text{Normal}(0, 2) \\
\bar{\beta} &\sim \text{Normal}(0, 0.5) \\
\lambda &\sim \text{Normal}(0, 0.1) \\
\kappa &\sim \text{Exponential}(5) \\
\mathbf{R} &\sim \text{LKJCorr}(1) \\
\tau_\alpha, \tau_\beta, \tau_P, \tau_G, \tau_L, \tau_R &\sim \text{Exponential}(4)
\end{aligned}
$$

where ζ parameters represent ordinal intercept cutpoints, $\bar{\beta}$ represents the slope fixed

effect, other α and β parameters represent random intercepts and slopes, τ parameters represent standard deviations for random effects, $\mathbf{R}$ represents the correlation matrix for the item random effects, and λ, κ, and z represent the mean, standard deviation, and standardised latent values for the relational mobility measurement error model.

In *brms*, this model is written as follows:

```
brm(
  formula = Trust ~ 1 + me(RML, RML_SE, gr = iso) +
    (1 + RML | group) + (1 | id) + (1 | gr(iso1, cov = lingCov)) +
    (1 | gr(iso2, cov = geoCov)) + (1 | iso3),
  data = d, family = cumulative,
  prior = c(prior(normal(0, 2), class = Intercept),
            prior(normal(0, 0.5), class = b),
            prior(exponential(4), class = sd),
            prior(normal(0, 0.1), class = meanme),
            prior(exponential(5), class = sdme))
  )
```

Supplementary Figures

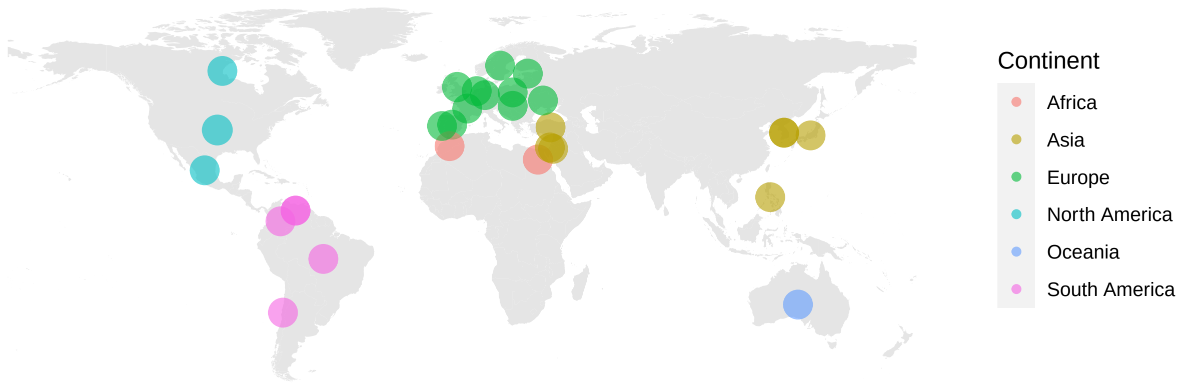

Figure S1. *Countries sampled in the final dataset for Study 1.* Data from the Global Preferences Survey. Point sizes indicate relative numbers of participants sampled in each country.

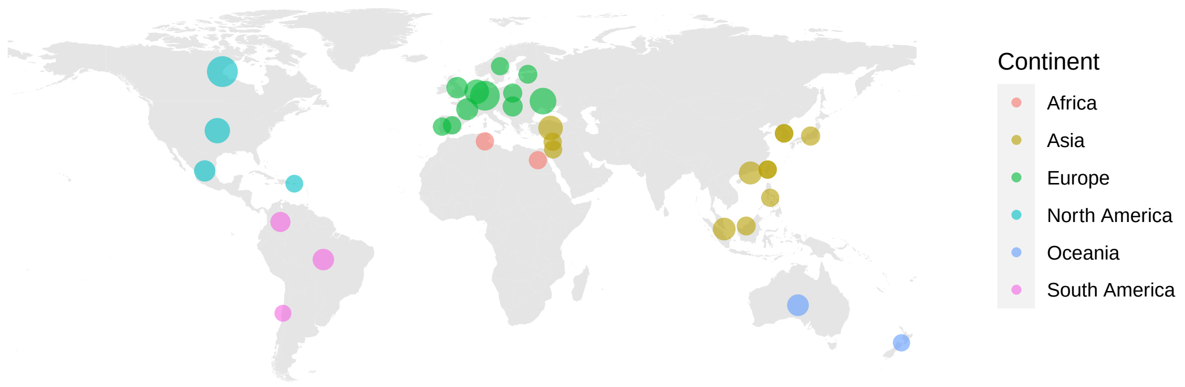

Figure S2. Countries sampled in the final dataset for Study 2. Data from the World Values Survey and European Values Survey. Point sizes indicate relative numbers of participants sampled in each country.

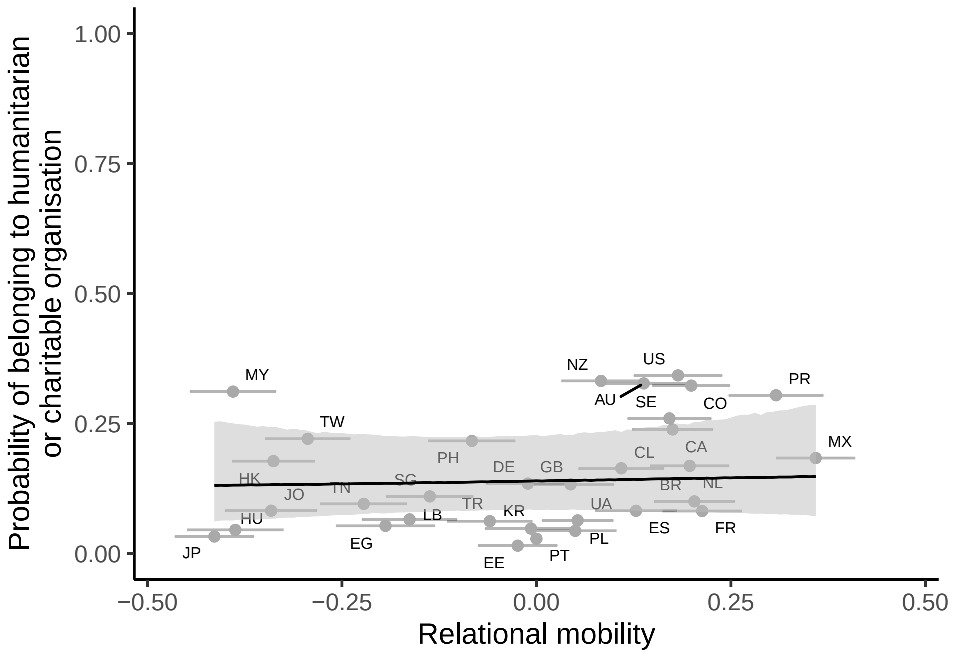

Figure S3. *Posterior predictions from a Bayesian multilevel logistic regression predicting charitable organisation membership from country-level relational mobility, controlling for environmental harshness and subsistence style.* The line and shaded area indicate the median posterior regression line and 95% credible intervals. Points indicate the proportion of individuals belonging to charitable organisations on the y-axis and relational mobility scores on the x-axis, for each of the 32 countries, with error bars representing +/- 1 standard error. Letters represent country ISO codes.

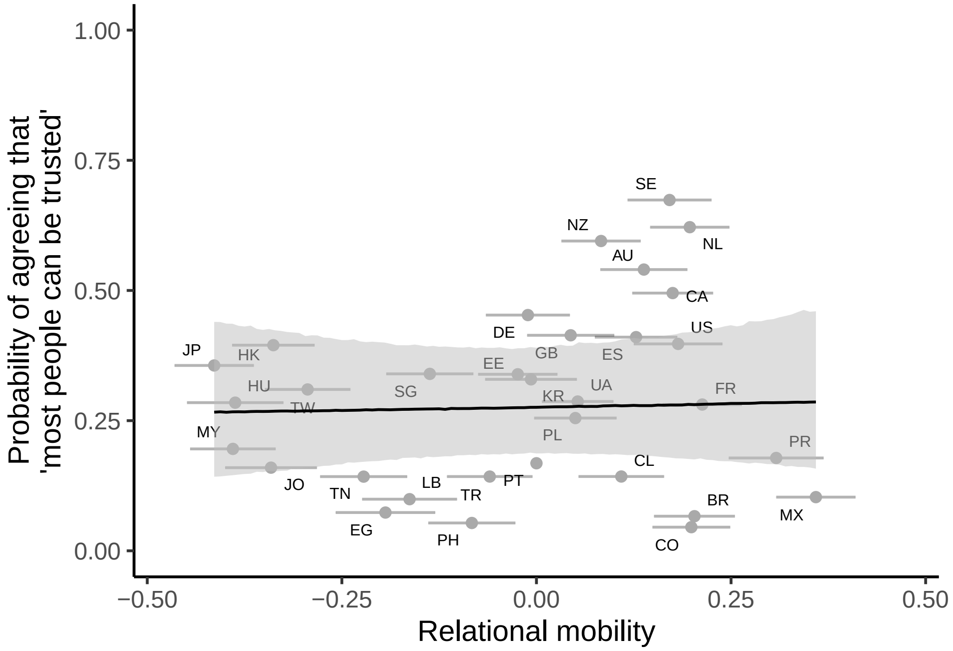

Figure S4. Posterior predictions from a Bayesian multilevel logistic regression predicting generalised trust from country-level relational mobility, controlling for environmental harshness and subsistence style. The line and shaded area indicate the median posterior regression line and 95% credible intervals. Points indicate the proportion of individuals stating that "most people can be trusted" on the y-axis and relational mobility scores on the x-axis, for each of the 32 countries, with error bars representing +/- 1 standard error. Letters represent country ISO codes.

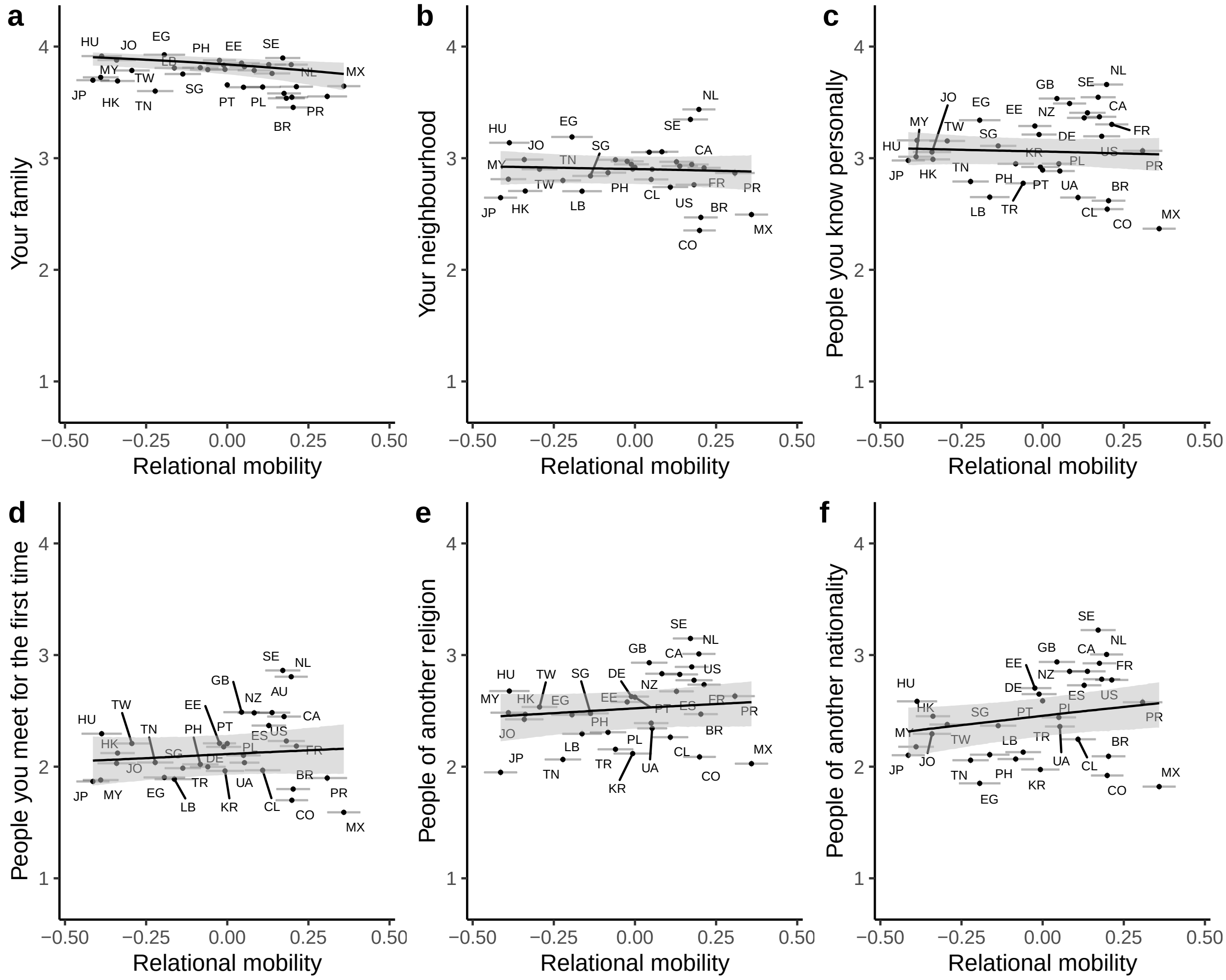

Figure S5. Posterior predictions from a Bayesian multilevel ordinal regression predicting trust in specific groups from country-level relational mobility, controlling for environmental harshness and subsistence style. Lines and shaded areas indicate median posterior regression lines and 95% credible intervals. Points indicate average trust and relational mobility scores for each of the 32 countries, with error bars representing +/- 1 standard error. Letters represent country ISO codes.

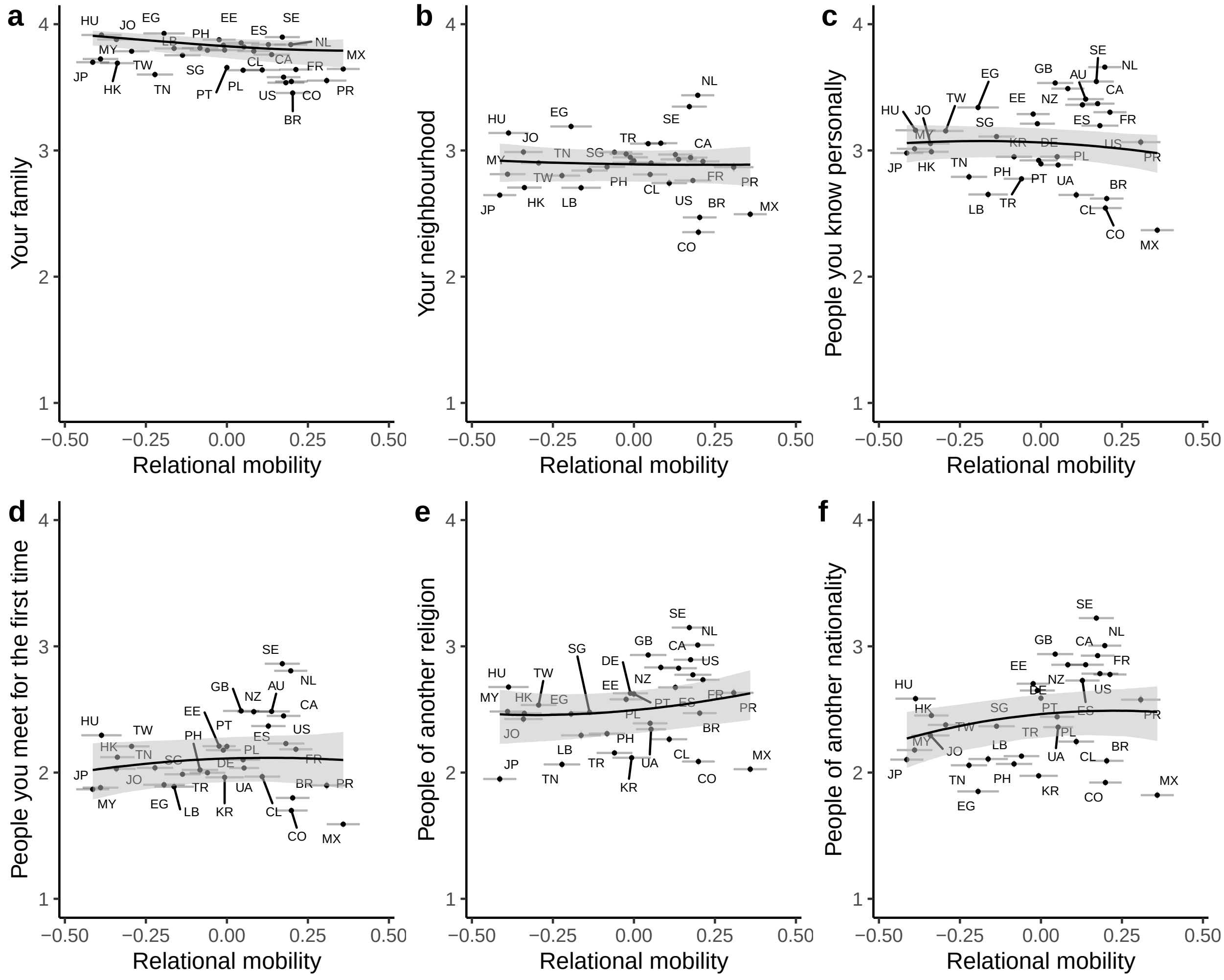

Figure S6. *Posterior predictions from a Bayesian multilevel ordinal regression predicting trust in specific groups from country-level relational mobility, controlling for environmental harshness and subsistence style and including a quadratic effect for relational mobility.* Lines and shaded areas indicate median posterior regression lines and 95% credible intervals. Points indicate average trust and relational mobility scores for each of the 32 countries, with error bars representing +/- 1 standard error. Letters represent country ISO codes.

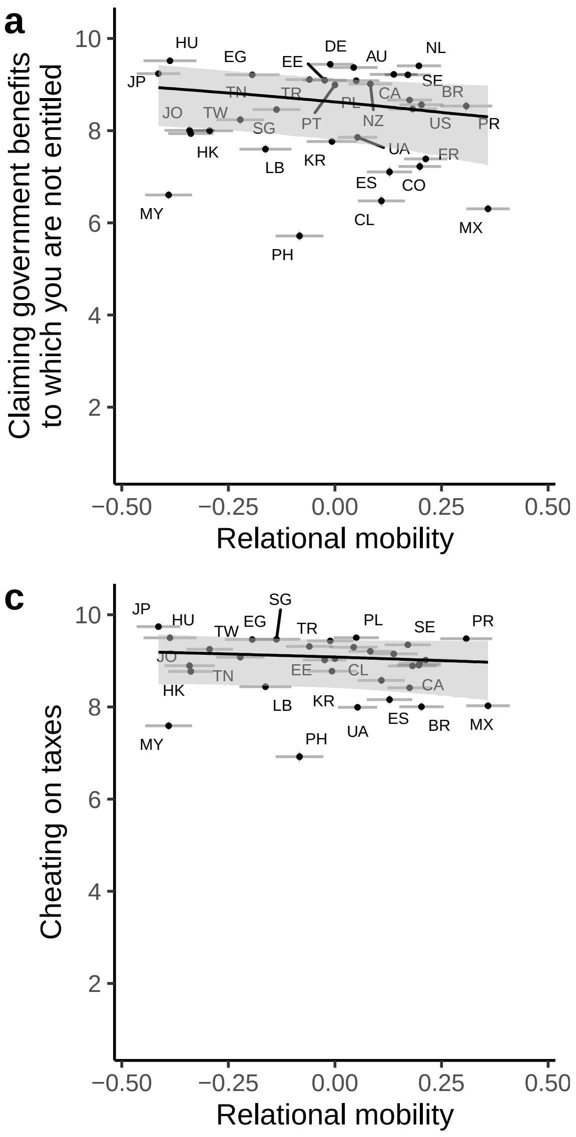

Figure S7. *Posterior predictions from a Bayesian multilevel ordinal regression predicting moral justifiability of different scenarios from country-level relational mobility, controlling for environmental harshness and subsistence style.* Higher numbers on the y-axis indicate *lower* justifiability ratings for each scenario. Lines and shaded areas indicate median posterior regression lines and 95% credible intervals. Points indicate average justifiability (reversed) and relational mobility scores for each of the 32 countries, with error bars representing +/- 1 standard error. Letters represent country ISO codes.

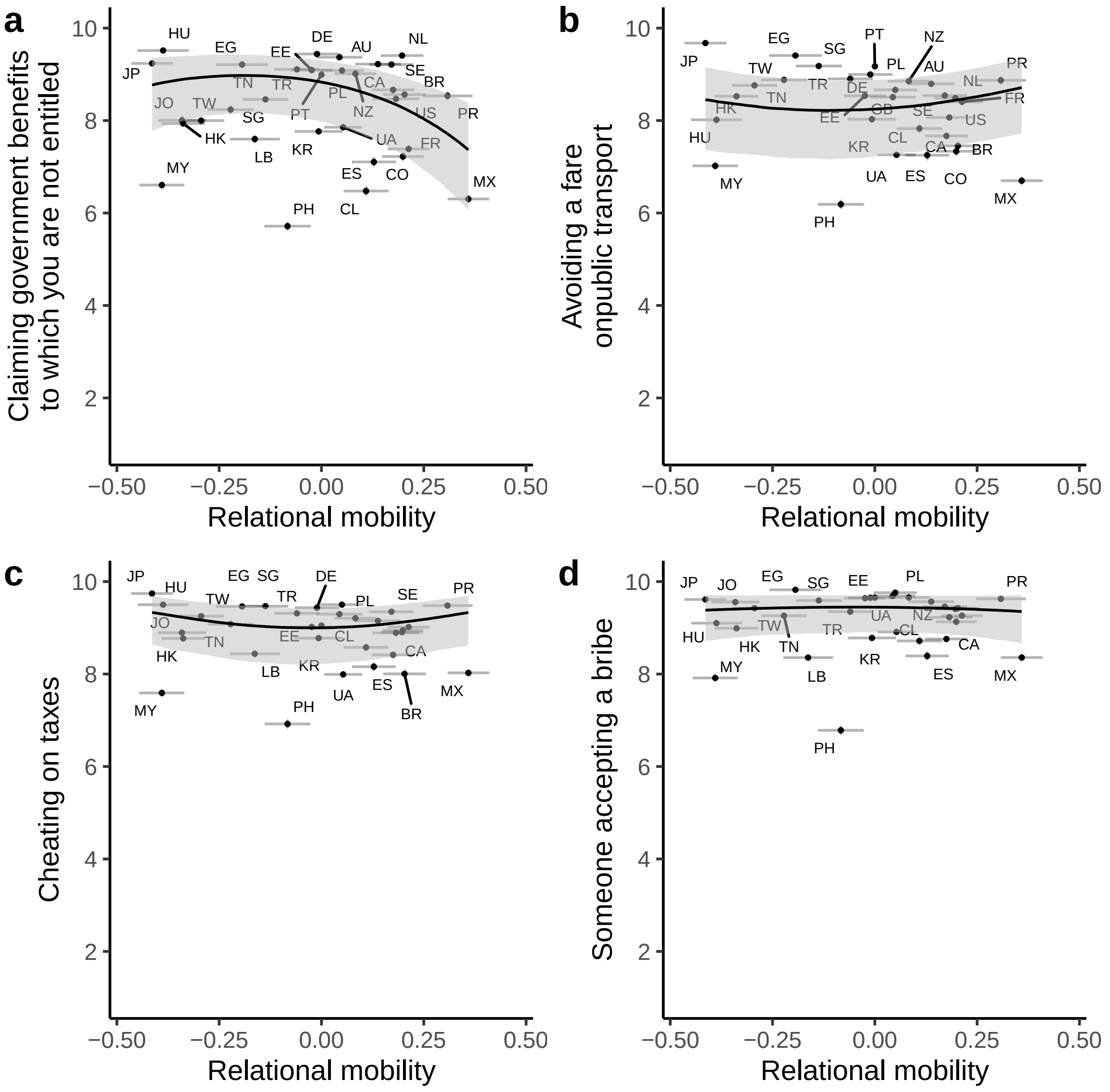

Figure S8. Posterior predictions from a Bayesian multilevel ordinal regression predicting moral justifiability of different scenarios from country-level relational mobility, controlling for environmental harshness and subsistence style and including a quadratic effect for relational mobility. Higher numbers on the y-axis indicate *lower* justifiability ratings for each scenario. Lines and shaded areas indicate median posterior regression lines and 95% credible intervals. Points indicate average justifiability (reversed) and relational mobility scores for each of the 32 countries, with error bars representing +/- 1 standard error. Letters represent country ISO codes.

Supplementary Tables

Table S1

Raw national-level data from Study 1. Mean averages are reported for prosocial measures from the Global Preferences Survey. SE = standard error for relational mobility score.

Country	Positive reciprocity	Trust	Altruism	Relational mobility	SE
Australia	0.07	0.38	0.18	0.14	0.06
Brazil	0.36	-0.04	0.46	0.20	0.05
Canada	0.22	0.31	0.24	0.17	0.05
Chile	0.07	-0.07	-0.06	0.11	0.06
Colombia	0.16	0.03	0.08	0.20	0.05
Egypt	0.55	0.61	0.64	-0.19	0.06
Estonia	-0.47	0.14	-0.57	-0.02	0.05
France	-0.12	-0.20	-0.16	0.21	0.05
Germany	-0.04	-0.11	0.01	-0.01	0.05
Hungary	-0.06	0.56	-0.54	-0.39	0.06
Israel	-0.02	-0.04	-0.33	0.09	0.06
Japan	-0.19	-0.49	-0.20	-0.41	0.05
Jordan	0.32	0.41	0.22	-0.34	0.06
Mexico	-1.11	-0.40	-0.84	0.36	0.05
Morocco	0.55	-0.10	0.55	-0.14	0.06
Netherlands	-0.09	0.34	-0.13	0.20	0.05
Philippines	0.19	0.31	0.38	-0.08	0.06
Poland	-0.13	-0.12	-0.34	0.05	0.05
Portugal	0.23	0.13	0.07	0.00	0.00
South Korea	-0.12	-0.05	0.42	-0.01	0.06
Spain	0.33	0.26	-0.11	0.13	0.05
Sweden	0.01	0.35	-0.15	0.17	0.05
Turkey	-0.42	0.08	-0.25	-0.06	0.06
UK	-0.05	0.25	0.03	0.04	0.06
Ukraine	0.16	-0.08	-0.10	0.05	0.05
USA	0.16	0.23	0.38	0.18	0.06
Venezuela	0.02	0.12	0.08	0.23	0.05

Table S2

Measurement invariance results for the prosociality measures from the Global Preferences Survey. Across nations, the analyses tested the measurement invariance of the factor structure for a single factor with loadings from altruism, positive reciprocity, and trust. Thresholds for good fit: RMSEA < 0.08; SRMR < 0.08; CFI > 0.95 (Hu & Bentler, 1999; MacCallum et al., 1996).

Model	RMSEA	CFI	SRMR
Configural invariance	0.00	1.00	0.00
Metric invariance	0.05	0.98	0.02
Scalar invariance	0.16	0.44	0.09

Table S3

Results from power analysis simulations. For each analysis, we simulated multiple datasets with various effect sizes (slopes) for relational mobility and, as a measure of power, determined the proportion of models fitted to these datasets that returned significantly positive slopes ($p < 0.05$). We manipulated the effect sizes until analyses returned around 80% power. For effect size thresholds in regression, see Funder & Ozer (2019). For effect size thresholds in logistic regression, see Chen, Cohen, and Chen (2010).

Outcome	Model	Slope	Effect size	Power	Lower 95%	Upper 95%
GPS Prosociality	Multilevel regression	0.28	Medium	0.83	0.80	0.87
WVS Charitable	Multilevel logistic regression	0.59	Small	0.80	0.77	0.82
WVS Trust	Multilevel logistic regression	0.58	Small	0.84	0.81	0.86
WVS Trust Groups	Multilevel regression	0.20	Small	0.79	0.70	0.87
WVS Justify	Multilevel regression	0.25	Medium	0.81	0.72	0.88

Table S4

Posterior slopes from models with quadratic effects of relational mobility.

Outcome	Parameter	Linear slope	Quadratic slope
GPS Prosociality	Population-level	b = -0.01, 95% CI [-0.20, 0.17]	b = -0.01, 95% CI [-0.21, 0.19]
	RE: Altruism	b = 0.41, 95% CI [-0.07, 0.84]	b = 0.02, 95% CI [-0.28, 0.39]
	RE: Positive reciprocity	b = -0.07, 95% CI [-0.54, 0.37]	b = -0.07, 95% CI [-0.51, 0.20]
	RE: Trust	b = -0.64, 95% CI [-1.11, -0.20]	b = -0.03, 95% CI [-0.39, 0.29]
WVS Charitable	Population-level	b = 0.19, 95% CI [-1.34, 1.73]	b = 0.09, 95% CI [-1.77, 2.01]
WVS Trust	Population-level	b = 0.07, 95% CI [-1.39, 1.56]	b = -0.11, 95% CI [-1.93, 1.71]
WVS Trust Groups	Population-level	b = 0.02, 95% CI [-0.90, 0.95]	b = -0.06, 95% CI [-1.06, 0.94]
	RE: Another nationality	b = 0.72, 95% CI [-0.25, 1.74]	b = -1.57, 95% CI [-2.94, -0.19]
	RE: Another religion	b = 0.76, 95% CI [-0.23, 1.81]	b = 1.23, 95% CI [-0.16, 2.70]
	RE: Know personally	b = -0.54, 95% CI [-1.53, 0.48]	b = -1.43, 95% CI [-2.87, -0.01]
	RE: Meet first time	b = 0.25, 95% CI [-0.74, 1.27]	b = -0.95, 95% CI [-2.37, 0.48]
	RE: Family	b = -1.14, 95% CI [-2.12, -0.10]	b = 1.52, 95% CI [0.03, 2.96]
	RE: Neighbourhood	b = -0.16, 95% CI [-1.14, 0.88]	b = 0.36, 95% CI [-1.04, 1.79]
WVS Justify	Population-level	b = -0.20, 95% CI [-1.12, 0.70]	b = 0.03, 95% CI [-0.92, 0.96]
	RE: Public transport	b = 0.53, 95% CI [-0.71, 1.71]	b = 2.50, 95% CI [-0.07, 5.04]
	RE: Cheat taxes	b = 0.20, 95% CI [-1.07, 1.41]	b = 3.64, 95% CI [1.10, 6.16]
	RE: Gov benefits	b = -2.02, 95% CI [-3.32, -0.82]	b = -5.33, 95% CI [-7.91, -2.81]
	RE: Accept bribe	b = -0.10, 95% CI [-1.36, 1.08]	b = -1.08, 95% CI [-3.65, 1.46]

Table S5

Raw national-level data from Study 2. Mean averages are reported for prosocial measures from the World Values Survey. SE = standard error for relational mobility score.

Country	CharOrg	Trust	TruFam	TruNeigh	TruKnow	TruMeet	TruRel	TruNat	JusGovBen	JusFare	JusTax	JusBribe	RelMob	SE
Australia	0.33	0.54	3.76	2.93	3.41	2.48	2.83	2.85	9.22	8.79	9.15	9.56	0.14	0.06
Brazil	0.10	0.07	3.45	2.47	2.62	1.80	2.47	2.09	8.56	7.45	8.00	9.43	0.20	0.05
Canada	0.24	0.50	3.58	2.94	3.37	2.45	2.89	2.93	8.66	7.67	8.42	8.76	0.17	0.05
Chile	0.16	0.14	3.64	2.74	2.65	1.97	2.26	2.25	6.48	7.83	8.58	8.72	0.11	0.06
Colombia	0.32	0.05	3.55	2.35	2.54	1.70	2.09	1.92	7.22	7.34	8.95	9.13	0.20	0.05
Egypt	0.05	0.07	3.93	3.19	3.34	1.90	2.46	1.85	9.21	9.41	9.46	9.82	-0.19	0.06
Estonia	0.02	0.34	3.88	2.97	3.29	2.21	2.58	2.70	9.09	8.54	9.02	9.64	-0.02	0.05
France	0.08	0.28	3.64	2.91	3.30	2.18	2.73	2.78	7.39	8.41	9.02	9.26	0.21	0.05
Germany	0.13	0.45	3.83	2.95	3.21	2.18	2.63	2.65	9.44	9.00	9.43	9.65	-0.01	0.05
Hong Kong	0.18	0.39	3.69	2.71	2.99	2.12	2.47	2.45	7.94	8.53	8.77	8.99	-0.34	0.05
Hungary	0.05	0.28	3.91	3.14	3.16	2.30	2.68	2.59	9.52	8.02	9.50	9.10	-0.39	0.06
Japan	0.03	0.36	3.70	2.65	2.98	1.87	1.95	2.10	9.24	9.68	9.74	9.61	-0.41	0.05
Jordan	0.08	0.16	3.88	2.99	3.05	2.03	2.42	2.29	8.01		8.89	9.56	-0.34	0.06
Lebanon	0.07	0.10	3.81	2.70	2.65	1.89	2.29	2.11	7.60		8.44	8.36	-0.16	0.06
Malaysia	0.31	0.20	3.72	2.81	3.01	1.88	2.48	2.18	6.60	7.02	7.59	7.92	-0.39	0.06
Mexico	0.18	0.10	3.65	2.49	2.37	1.59	2.03	1.82	6.30	6.70	8.03	8.36	0.36	0.05
Netherlands	0.17	0.62	3.84	3.44	3.66	2.81	3.01	3.01	9.41	8.49	8.91	9.41	0.20	0.05
New Zealand	0.33	0.60	3.79	3.06	3.49	2.48	2.83	2.85	9.01	8.85	9.21	9.66	0.08	0.05
Philippines	0.22	0.05	3.81	2.87	2.95	2.02	2.31	2.07	5.72	6.19	6.92	6.78	-0.08	0.06
Poland	0.04	0.25	3.64	2.81	2.95	2.10	2.39	2.44	9.08	8.66	9.50	9.76	0.05	0.05
Portugal	0.03	0.17	3.66	2.92	2.89	2.21	2.62	2.59	8.99	9.17	9.05	9.65	0.00	0.00
Puerto Rico	0.30	0.18	3.55	2.87	3.07	1.90	2.63	2.58	8.53	8.87	9.48	9.63	0.31	0.06
Singapore	0.11	0.34	3.75	2.84	3.11	1.99	2.48	2.37	8.46	9.18	9.47	9.59	-0.14	0.06
South Korea	0.05	0.33	3.80	2.90	2.92	1.96	2.12	1.97	7.76	8.03	8.78	8.78	-0.01	0.06
Spain	0.08	0.41	3.84	2.97	3.36	2.37	2.67	2.73	7.10	7.25	8.16	8.39	0.13	0.05
Sweden	0.26	0.67	3.90	3.35	3.55	2.86	3.15	3.22	9.21	8.54	9.35	9.46	0.17	0.05
Taiwan	0.22	0.31	3.79	2.90	3.15	2.21	2.53	2.38	8.00	8.76	9.25	9.43	-0.29	0.06
Tunisia	0.10	0.14	3.60	2.80	2.79	2.04	2.06	2.06	8.24	8.88	9.08	9.26	-0.22	0.06
Turkey	0.06	0.14	3.79	2.99	2.78	2.00	2.16	2.13	9.11	8.90	9.31	9.35	-0.06	0.06
UK	0.13	0.41	3.85	3.05	3.53	2.49	2.93	2.94	9.37	8.51	9.30	9.69	0.04	0.06
Ukraine	0.06	0.29	3.82	2.90	2.88	2.04	2.34	2.36	7.86	7.26	7.99	8.91	0.05	0.05
USA	0.34	0.40	3.54	2.76	3.20	2.23	2.77	2.78	8.47	8.07	8.89	9.23	0.18	0.06

Table S6

Measurement invariance results for the measures of trust in different groups from the World Values Survey. Across nations, the analyses tested the measurement invariance of the factor structure for two factors: (1) trust in your family, people in your neighbourhood, and people you know personally, and (2) trust in people you meet for the first time, people of another nationality, and people of another religion. Thresholds for good fit: RMSEA < 0.08; SRMR < 0.08; CFI > 0.95 (Hu & Bentler, 1999; MacCallum et al., 1996).

Model	RMSEA	CFI	SRMR
Configural invariance	0.10	0.95	0.04
Metric invariance	0.09	0.94	0.06
Scalar invariance	0.14	0.83	0.09

Table S7

Measurement invariance results for the moral justifiability measures from the World Values Survey. Across nations, the analyses tested the measurement invariance of the factor structure for a single factor with loadings from all four items: claiming government benefits, avoiding public transport fare, cheating on taxes, and accepting a bribe. Thresholds for good fit: RMSEA < 0.08; SRMR < 0.08; CFI > 0.95 (Hu & Bentler, 1999; MacCallum et al., 1996).

Model	RMSEA	CFI	SRMR
Configural invariance	0.14	0.96	0.03
Metric invariance	0.13	0.93	0.07
Scalar invariance	0.17	0.79	0.11

Supplementary References

Chen, H., Cohen, P., & Chen, S. (2010). How big is a big odds ratio? Interpreting the magnitudes of odds ratios in epidemiological studies. *Communications in Statistics — Simulation and Computation*, *39*(4), 860–864. https://doi.org/10.1080/03610911003650383

Cronk, L., Berbesque, C., Conte, T., Gervais, M., Iyer, P., McCarthy, B., Sonkoi, D., Townsend, C., & Aktipis, A. (2019). Managing risk through cooperation: Need-based transfers and risk pooling among the societies of the Human Generosity Project. In L. R. Lozny & T. H. McGovern (Eds.), *Global perspectives on long term community resource management* (pp. 41–75). Springer International Publishing. https://doi.org/10.1007/978-3-030-15800-2_4

Eberhard, D. M., Simons, G. F., & Fennig, C. D. (Eds.). (2018). *Ethnologue: Languages of the world* (Twenty-first). SIL International.

Eff, E. A. (2008). Weight matrices for cultural proximity: Deriving weights from a language phylogeny. *Structure and Dynamics*, *3*(2). https://doi.org/10.5070/SD932003296

Funder, D. C., & Ozer, D. J. (2019). Evaluating effect size in psychological research: Sense and nonsense. *Advances in Methods and Practices in Psychological Science*, *2*(2), 156–168. https://doi.org/10.1177/2515245919847202

Hammarström, H., Forkel, R., Haspelmath, M., & Bank, S. (2017). *Glottolog 3.0.* Max Planck Institute for the Science of Human History. https://doi.org/10.5281/zenodo.4061162

Hu, L. T., & Bentler, P. M. (1999). Cutoff criteria for fit indexes in covariance structure analysis: Conventional criteria versus new alternatives. *Structural Equation Modeling*, *6*(1), 1–55. https://doi.org/10.1080/10705519909540118

MacCallum, R. C., Browne, M. W., & Sugawara, H. M. (1996). Power analysis and determination of sample size for covariance structure modeling. *Psychological Methods*,

1(2), 130–149. https://doi.org/10.1037/1082-989x.1.2.130

Talhelm, T., Zhang, X., Oishi, S., Shimin, C., Duan, D., Lan, X., & Kitayama, S. (2014). Large-scale psychological differences within china explained by rice versus wheat agriculture. *Science*, *344*(6184), 603–608. https://doi.org/10.1126/science.1246850

Thomson, R., Yuki, M., Talhelm, T., Schug, J., Kito, M., Ayanian, A. H., Becker, J. C., Becker, M., Chiu, C.-y., Choi, H.-S., Ferreira, C. M., Fülöp, M., Gul, P., Houghton-Illera, A. M., Joasoo, M., Jong, J., Kavanagh, C. M., Khutkyy, D., Manzi, C., … Visserman, M. L. (2018). Relational mobility predicts social behaviors in 39 countries and is tied to historical farming and threat. *Proceedings of the National Academy of Sciences*, *115*(29), 7521–7526. https://doi.org/10.1073/pnas.1713191115

Villemereuil, P. D., & Nakagawa, S. (2014). General quantitative genetic methods for comparative biology. In *Modern phylogenetic comparative methods and their application in evolutionary biology* (pp. 287-303). Springer, Berlin, Heidelberg.
